# Modeling Non-inherited Antibiotic Resistance

**DOI:** 10.1007/s11538-012-9731-3

**Published:** 2012-05-19

**Authors:** M. C. J. Bootsma, M. A. van der Horst, T. Guryeva, B. H. ter Kuile, O. Diekmann

**Affiliations:** 1 Faculty of Science, Department of Mathematics, Utrecht University, Budapestlaan 6, P.O. Box 80010, 3584 CD Utrecht, The Netherlands; 2 Julius Center for Health Research & Primary Care, University Medical Center Utrecht, Heidelberglaan 100, P.O. Box 85500, 3508 GA Utrecht, The Netherlands; 3 Laboratory for Molecular Biology and Microbial Food Safety, Swammerdam Institute of Life Sciences, University of Amsterdam, Nieuwe Achtergracht 166, 1018 WV Amsterdam, The Netherlands; 4 Office for Risk Assessment, Netherlands Food and Consumer Product Safety Authority, Catharijnesingel 59, 3511 GG Utrecht, The Netherlands

**Keywords:** *E. coli*, Mathematical model, Markov Chain Monte Carlo, Non-inherited resistance

## Abstract

A mathematical model is presented for the increase and decrease of non-inherited antibiotic resistance levels in bacteria. The model is applied to experimental data on *E. coli* exposed to amoxicillin or tetracyclin in different concentrations. The parameters of the model are estimated using a Monte Carlo Markov Chain method. The model accurately describes build-up and decline of antibiotic resistance caused by physiological adaptations as long as no genetic changes have occurred. The main conclusion of the analysis is that short time periods are sufficient to re-obtain low MIC-values after long-lasting exposure to these antibiotics.

## Introduction

In recent years, the increasing abundance of antimicrobial resistance has caused mounting costs in the health care sector. Treatment of patients infected by resistant pathogens such as methicillin resistant *Staphylococcus aureus* (MRSA) or carriers of the extended spectrum beta-lactamases (ESBL) gene, costs between $6,000 and $50,000 more than therapy of those suffering from susceptible variants (Maragakis et al. 2008; Slama 2008). The stage has been reached that the medicines of choice are no longer effective and that infections with these multi-resistant micro-organisms can only be treated with more expensive or more toxic drugs. In some cases, even none of the available antibiotics has sufficient effect.

The problem is not limited to human health care, the veterinary sector is confronted with the same problem (Mevius et al. 2010). The nature and evolution of tolerance and resistance of microbes against antibiotics has been documented in some 200,000 scientific articles (Davies and Davies 2010). Three types of mechanisms can cause a rise of resistance: (1) adaptation of the regular cellular machinery, (2) mutations that make the target insensitive, and (3) transfer of so-called resistance genes (Harbottle et al. 2006). Once any of these has occurred, further exposure to the antibiotic will select for the specific trait. Despite the detailed knowledge about mechanisms and epidemiology of resistance, the rate of appearance upon exposure to antibiotics and the rate of decline when the selective pressure is removed, are not known quantitatively. Build-up and decline of antibiotic resistance depend on a large number of factors and the rates are likely to differ between various combinations of microbial species and antibiotics. Important components are: the initial acquisition of resistance by a single cell, or at most a few cells, and the subsequent positive selection of these resistant cells. Acquisition can be by any of the three events mentioned above. While the second two are by nature chance events, the first is usually gradual, is most likely to occur in more than one cell simultaneously, and is therefore more predictable.

The experimental data in this article were obtained in the laboratory using *E. coli* as a model organism and tetracyclin, amoxicillin, and enrofloxacin as antibiotics (see Sect. 2 for details). An *E. coli*-strain was grown in known, but time-varying, concentrations of an antibiotic in a well-shaken medium. At several time points, samples of the *E. coli* population were exposed to different dilutions of antibiotics in a 96 well-plate. The minimal inhibitory concentration (MIC) was the lowest concentration of antibiotics in the 96-well plate at which no growth was observed after 24 hours.

As explained below, for tetracyclin, physiological adaptation seems a plausible explanation of the lab experiments while this is clearly not the case for enrofloxacin. For amoxicillin, physiological adaptation seems to be present but inherited resistance may have developed as well.

In the study reported here, we have developed a model for the acquisition of antibiotic resistance by means of physiological adaptation. Several mathematical models have been developed to describe the likelihood of development of heritable resistance, e.g. a mutation, and its subsequent spread in a population; see e.g. Abel zur Wiesch et al. (2011), Day and Bonduriansky (2011). To our knowledge, no mathematical models have been developed that describe the mechanisms underlying physiological adaptation.

For tetracyclin, the model that was developed allows reasonably accurate predictions of the actual development of resistance once the relevant parameters have been determined. These models can be used by public health services involved in regulating the use of antibiotics in the agricultural sector to predict the effects of measures under consideration. Indeed, if animals receive antibiotics, there may be residues of these antibiotics present in the animals at the moment of slaughtering and this has led to the ban of certain classes of antibiotics in a time-window before slaughter. However, the antibiotics prescribed earlier, may cause an increase in the resistance level in bacteria and this increased resistance level may still be present at the moment of slaughter. Because the meat is for human consumption, this may lead to cases of gastroenteritis in humans that are hard to treat.

## Experimental Procedures

**Fig. 1:**
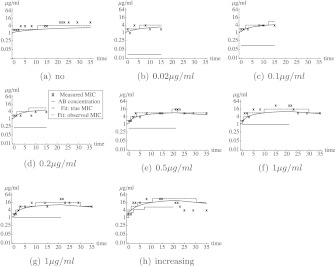
Observed MIC values in the tetracyclin lab experiments and the best model fit. Each figure corresponds to a different experiment. In (**a**), the *E. coli* were grown in absence of antibiotics. In (**b**)–(**d**), the *E. coli* grew in the presence of a constant concentration of tetracyclin from day 0 to day 15. In (**e**)–(**g**), the *E. coli* grew in the presence of a constant concentration of tetracyclin from day 0 to day 21. Afterward, the *E. coli* grew in absence of tetracyclin. In (**h**), the concentration of tetracyclin was stepwise increased. The *black crosses* correspond to the data from the experiments. The *blue curve* corresponds to the fit, the *dashed blue* step function correspond to the MIC-value that would have been observed if there would be no measurement error. The *red lines* correspond to the concentration of tetracycline to which the *E. coli* cells are exposed (Color figure online)

**Fig. 2:**
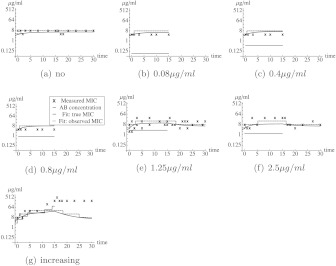
Observed MIC values in the amoxicillin lab experiments and the best model fit. Each figure corresponds to a different experiment. In (**a**), the *E. coli* were grown in absence of antibiotics. In (**b**)–(**d**), the *E. coli* grew in the presence of a constant concentration of amoxicillin from day 0 to day 15. In (**e**)–(**f**), the *E. coli* grew in the presence of a constant concentration of amoxicillin from day 0 to day 15. Afterward, the *E. coli* grew in absence of amoxicillin. In (**h**), the concentration of amoxicillin was stepwise increased. The *black crosses* correspond to the data from the experiments. The *blue curve* corresponds to the fit, the *dashed blue* step function correspond to the MIC-value that would have been observed if there would be no measurement error. The *red lines* correspond to the concentration of amoxicillin to which the *E. coli* cells are exposed. The data in (**g**) were not used to determine the best fit, as the resistance seems to be inherited in this experiment (Color figure online)

**Fig. 3:**
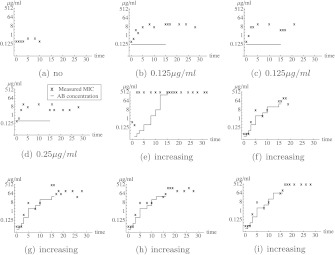
Observed MIC values in the enrofloxacin lab experiments. Each figure corresponds to a different experiment. In (**a**), the *E. coli* were grown in absence of antibiotics. In (**b**)–(**d**), the *E. coli* grew in the presence of a constant concentration of enrofloxacin from day 0 to day 15. In (**e**)–(**i**), the *E. coli* grew from day 0–15 in the presence of a stepwise increasing concentration of amoxicillin. After day 15, the cells grew in absence of enrofloxacin. The *black crosses* correspond to the data from the experiments. The *red lines* correspond to the concentration of enrofloxacin to which the *E. coli* cells are exposed. No model fit is presented as the resistance seems inherited (Color figure online)

Growing *E. coli* MG 1655 cells were exposed to constant or step-wise increasing concentrations of amoxicillin, tetracycline, or enrofloxacin in defined minimal mineral medium containing 55 mM glucose at a temperature of 37 ^∘^ Celsius, with a pH of 7.0 and a buffer of 15.6 g/l Na_2_H_2_PO_4_ (Van der Horst et al. 2011). The *E. coli* MG 1655 come from the freezer and are grown for 24 hours before the first measurement. Cells growing at a normal rate were transferred after 24 hours. Cells in the step-wise increasing experiments that were growing slowly due to the presence of antibiotics were transferred once a sufficient density had been reached. When they grew at a normal or almost normal rate, a new incubation at double the concentration was started. This cycle was repeated as often as feasible. After 15 or 21 days of growth in the presence of antibiotics, the cells were grown in an antibiotic-free medium. The value of the minimal inhibitory concentration (MIC) was determined as described earlier (Schuurmans et al. 2009) by exposing samples of the bacterial population to serial two-fold dilutions of the antibiotic in a 96-well plate. The optical density (at 600 nm) of cells at the start of the MIC measurement was approximately 0.05. The MIC-value was defined as the lowest concentration of antibiotics such that the optical density after 24 hours was below 0.2. In Figs. 1, 2, and 3 the observed MIC-values are shown for the experiments with tetracyclin, amoxicillin, and enroflaxacin, respectively. These figures also show the concentration of antibiotics to which the *E. coli* population was exposed as well as the model fit, which we will discuss later. For enrofloxacin, there was no gradual build-up of resistance, but big jumps in the MIC-value occurred (see Fig. 3). Even after enrofloxacin was removed, the MIC-value remained at a high level. This suggests that one or more mutations have occurred. Indeed, a single mutation in the *gyrA* gene of *E. coli* may cause high-level resistance against fluoroquinolones (Drlica et al. 2009). We will, therefore, focus on the other two types of antibiotics for which physiological adaptations are the more likely cause of changes in resistance. In case of the experiment with a step-wise increment in the amoxicillin-concentration (see Fig. 2(g)), the MIC-value did not decrease after the *E. coli* were grown in absence of amoxicillin. This suggests that also in this experiment an irreversible event happened. Therefore, this experiment was excluded from the data analysis.

## A Model for Non-inherited Resistance by Way of Efflux Pumps or Enzymes

Our first aim is to formulate a mathematical model that incorporates the following quantities:

- The concentration *A*_0_ of antibiotics outside the cells.
- The concentration *A* of antibiotics inside the cells or, for that matter, the density of antibiotic molecules attached to the cell wall.
- The concentration *E* of anti-antibiotic molecules, say efflux pumps or inactivating enzymes (the interpretation is less clear in the case of cells reacting by building their cell wall from different building blocks).

The meaning of the parameters is summarized in Table 1.

**Table 1:** Symbols used

Symbol	Interpretation
*λ*	Degradation rate of efflux pumps (enzymes)
*ξ*	Antibiotic diffusion coefficient
*ν*	Efficacy of efflux pumps (enzymes)
*c*_0_	Production rate of efflux pumps (enzymes) in absence of antibiotics in the cell
*c*_*M*_	Maximal increase in production rate of efflux pumps (enzymes)
*E*_0_	Initial concentration/density of efflux pumps (enzymes)
*M*_0_	Initial MIC-value
*A*_*c*_	Antibiotic concentration within cells at which cells stop growing
*A*_0_	Antibiotic concentration outside the cell
*A*_*h*_	Antibiotic concentration within the cell at which the production rate of efflux pumps (enzymes) is exactly between the minimum and maximum production rate
*σ*	Standard deviation of measurement/biological error
*κ*_1_	$A_{c}(\lambda+c_{0}\frac{\nu}{\xi})$
*κ*_2_	$\frac{\nu}{\xi}c_{M}A_{c}$
*κ*_3_	$\frac{A_{c}}{A_{h}}$

The model should describe quantitatively how

- changes of *A* relate to *A*_0_.
- A higher *A* leads to an increased production of *E*. (This can be interpreted as genes being switched on by the signal *A*.)
- *E* degrades in the course of time.
- *A* is broken down or eliminated by *E*.

From now on our formulation applies to efflux pumps (and hence requires some rephrasing when antibiotics are inactivated by enzymes). We assume the following. The concentration *A*_0_ of antibiotics outside the cells is constant, so we assume that the concentration of antibiotics outside the cells is not noticeably influenced by the amount taken up by the cells. The antibiotic concentration within a cell (*A*) increases (decreases) due to a higher (lower) concentration of antibiotics outside the cells (*A*_0_) by way of diffusion. The diffusion coefficient is denoted by *ξ*. The antibiotic concentration within a cell decreases due to the activity of efflux pumps. The more efflux pumps, the faster this process happens, and also, the higher the concentration of antibiotics within a cell, the more antibiotics the efflux pumps transport. We neglect the time it may take an efflux pump to transport an antibiotic molecule out of the cell. The efficacy of efflux pumps, i.e. the affinity for the antibiotic of the efflux pumps, is denoted by *ν*. Note that we do not take degradation of the antibiotic within a cell into account. If degradation is, for instance, slow as compared to the diffusion, this is a reasonable assumption.

We assume that the efflux pumps become ineffective at a constant rate *λ* and that their production rate depends on the antibiotic concentration within a cell, according to a Michaelis–Menten type function. This should occur via a regulation system in the cell, which we do not model explicitly. In absence of antibiotics in a cell, the production rate is *c*_0_ and the maximum production rate is *c*_0_+*c*_*M*_. The antibiotic concentration at which the production rate of efflux pumps is exactly between *c*_0_ and *c*_0_+*c*_*M*_ is denoted by *A*_*h*_. These assumptions lead to the following system of differential equations for the density of efflux pumps (*E*) and the antibiotic concentration (*A*) within the cell:

1 $$ \everymath{\displaystyle} \begin{array}{l} \frac{d}{dt}E=-\lambda E+c_0+\frac{c_{M}A}{A_h+A}\\[6pt] \frac{d}{dt}A=\xi(A_0-A)-\nu AE. \end{array} $$

The description in terms of ordinary differential equations requires that we neglect chance effects, i.e. we assume that there are many efflux pumps and antimicrobial molecules present. Moreover, we assume that all cells behave identical, e.g. that once a cell divides, both daughter cells have the same amount of antibiotics and efflux pumps and that a change in the ratio of cell volume and cell wall surface does not affect the dynamics. We assume that, once the concentration of antibiotics within the cell exceeds a critical level *A*_*c*_, the growth will stop. The external concentration of antibiotics (*A*_0_) needed to reach the critical antibiotic concentration within the cell is the MIC-value. In order for the MIC-value to have a clear meaning, the antibiotic concentration within the cell has to change rapidly after a change of the external concentration *A*_0_, i.e. we assume that the number of efflux pumps can be considered constant during the time the concentration in the cell changes as a result of a change in the external antibiotic concentration.

This is indeed the case, as a new steady concentration establishes within seconds after the external concentration of the antibiotic changes, whereas adaptation processes take a minimum of 20 minutes to start (Bolla et al. 2011). This implies that we can use a quasi-steady state approximation. The equilibrium antibiotic concentration within the cells for a constant number of efflux pumps is given by

2 $$ A=\frac{A_0}{1+\frac{\nu}{\xi} E} $$

and the critical external concentration, the MIC-value, is given by

3 $$ \hbox{MIC-value} = \biggl(1+\frac{\nu}{\xi} E\biggr) A_c. $$

If we substitute this equilibrium in the differential equation for the number of efflux pumps, we obtain

4 $$ \everymath{\displaystyle} \begin{array}{l} \frac{d}{dt}E=-\lambda E+c_0+\frac{c_M\frac{\xi}{\nu}A_0}{A_hE+\frac{\xi}{\nu}(A_0+A_h)} \\ E(t_0)=E_0 \end{array} $$

where *t*_0_ denotes the moment the external antibiotic concentration *A*_0_ is changed, e.g. the starting time of the experiment. Because the freezing of the *E. coli* may have damaged efflux pumps, the initial concentration of efflux pumps may be different from the equilibrium concentration efflux pumps in absence of antibiotics. In the experiments the MIC-value is determined, but the absolute number of efflux pumps and the antibiotic concentration within a cell are not measured and, therefore, unknown. For statistical purposes, it is therefore more useful to determine the time dynamics of the MIC-value, denoted by *M*(*t*).

If we define new parameters, $\kappa_{1}=A_{c}(\lambda+c_{0}\frac{\nu}{\xi})$, $\kappa_{2}=\frac{\nu}{\xi}c_{M}A_{c}$, $\kappa_{3}=\frac{A_{c}}{A_{h}}$, and $M_{0}=(1+\frac{\nu}{\xi} E_{0})A_{c}$, we can write the system as

5 $$ \everymath{\displaystyle} \begin{array}{l} \frac{d}{dt}M=-\lambda M+\kappa_1+\kappa_2\frac{\kappa_3A_0}{M+\kappa_3A_0}\\ M(t_0)=M_0. \end{array} $$

Based on observations of the MIC-value only, we can at most infer the values of the new parameters, *λ*, *κ*_1_, *κ*_2_, *κ*_3_, and *M*_0_ and not of all parameters of the original model. The positive equilibrium MIC-value, denoted by *M* ^+^, is given by

6 $$ M^+ = \frac{\kappa_1-A_0\kappa_3\lambda + \sqrt{((\kappa_1+A_0\kappa_3\lambda)^2+4A_0\kappa_2\kappa_3\lambda)}}{2\lambda}. $$

Note that *M* ^+^ depends on *A*_0_. When helpful we write *M* ^+^(*A*_0_) to reflect this dependence. For notational convenience, we also define the negative equilibrium MIC-value, which has no biological interpretation as

7 $$ M^- = \frac{\kappa_1-A_0\kappa_3\lambda - \sqrt{((\kappa_1+A_0\kappa_3\lambda)^2+4A_0\kappa_2\kappa_3\lambda)}}{2\lambda}. $$

By using the method of separation of variables, we can express *t* explicitly in terms of *M*. We define:

8 $$ f(M) = \frac{1}{\lambda}\biggl(\frac{\kappa_3A_0+M^-}{M^+-M^-}\log|M-M^-| - \frac{\kappa_3A_0+M^+}{M^+-M^-}\log|M-M^+|\biggr). $$

Using this definition, the solution of (5) can be written as

9 $$ t-t_0=f(M)-f(M_0). $$

Because there is no explicit inverse of the function *f*, we cannot obtain an explicit solution *M*(*t*) of (5), but numerically the inverse can be obtained very easily.

We want to use the model to extrapolate beyond situations for which we have experimental observations. In particular, we establish the relationship between certain input parameters (the cause) and output parameters (the effect). Apart from the resulting MIC-value, we also consider as an interesting output parameter the time *T*_*Q*_ it takes, after stopping the exposure to the antibiotic, before the MIC-value has decreased again to an acceptable level MIC_safe_. (Note that we have to choose this level; the idea is that when poultry is exposed to antibiotics, one wants to avoid that, by the time poultry comes into contact with consumers, the bacteria are still so antibiotic-tolerant that treatment may fail). To do this, we assume that the bacteria are exposed to a constant concentration *A*_0_ for a long period of time (so that the MIC-value is in its equilibrium value *M* ^+^(*A*_0_)). We then assume that the bacteria are no longer exposed to antibiotics. We define the time *T*_*Q*_ by the equation *M*(*T*_*Q*_)=MIC_safe_. If we use the differential equation of (5) with *A*_0_=0, we find that

10 $$ T_Q = \frac{1}{\lambda}\log\biggl\{ \frac{\kappa_1-\lambda M^+(A_0)}{\kappa_1-\lambda \mathrm{MIC}_{\mathrm{safe}}}\biggr\} $$

provided that MIC_safe_<*M* ^+^(*A*_0_) and $\mathrm{MIC}_{\mathrm{safe}}>\frac{\kappa_{1}}{\lambda}$, i.e. the MIC-value after long exposure to the antibiotic in concentration *A*_0_ exceeds MIC_safe_ and MIC_safe_ is higher than the MIC-value of cells never exposed to the antibiotic.

## Statistical Model

To determine the MIC-value at a certain time point during the experiments, a sample was taken and next grown in the presence of different concentrations (two-fold dilutions) of antibiotics. In the absence of measurement errors/biological fluctuations, an observed MIC-value of *y* implies that the true MIC-value is in the range $(\frac{y}{2},y)$. Of course, biological fluctuations and/or measurement errors may be present. We assumed each measurement error to be independent of all other measurements and the errors to be normally distributed with standard error *σ* on a logarithmic scale with base 2. If the model predicts that the MIC-value at the time of a certain measurement was *z*, the likelihood that we would observe an MIC-value of *y* is given by 
11


The likelihood of the entire data set for one antibiotic is the product of the likelihoods for a single measurement, so if we specify values for the unknown parameters *λ*, *κ*_1_, *κ*_2_, *κ*_3_, *M*_0_, and *σ*, we can calculate the likelihood of the observed data, i.e. *L*(data|*λ*,*κ*_1_,*κ*_2_,*κ*_3_,*M*_0_,*σ*).

We use a Monte Carlo Markov Chain (MCMC) (Gilks et al. 1996) algorithm to obtain credibility intervals for the 6 parameters. We use uninformative priors, i.e. uniform priors on (0; 1,000) for all parameters, with the additional condition that *E*_0_ is not larger than the equilibrium concentration of efflux pumps in absence of antibiotics, i.e. that the MIC-value is non-decreasing during the first 24 hours in absence of antibiotics. We use a Metropolis–Hastings updating scheme and 100,000,000 accepted MCMC updates after a burn-in period of 1,000,000 accepted updates. Convergence of the chain was checked by visual inspection.

## Results

### Tetracyclin Experiments

**Fig. 4:**
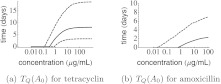
Time after long-lasting exposure to antibiotic in concentration *A*_0_ before the resistance level of *E. coli* drops below MIC_safe_. *Solid lines* correspond to the median value, *dashed lines* to the 95 % credibility intervals. MIC_safe_ is chosen to be 8 μg/ml for both tetracyclin and amoxicillin

**Fig. 5:**
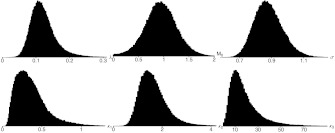
Histograms of the posterior distributions of the parameters for the tetracyclin experiments

**Fig. 6:**
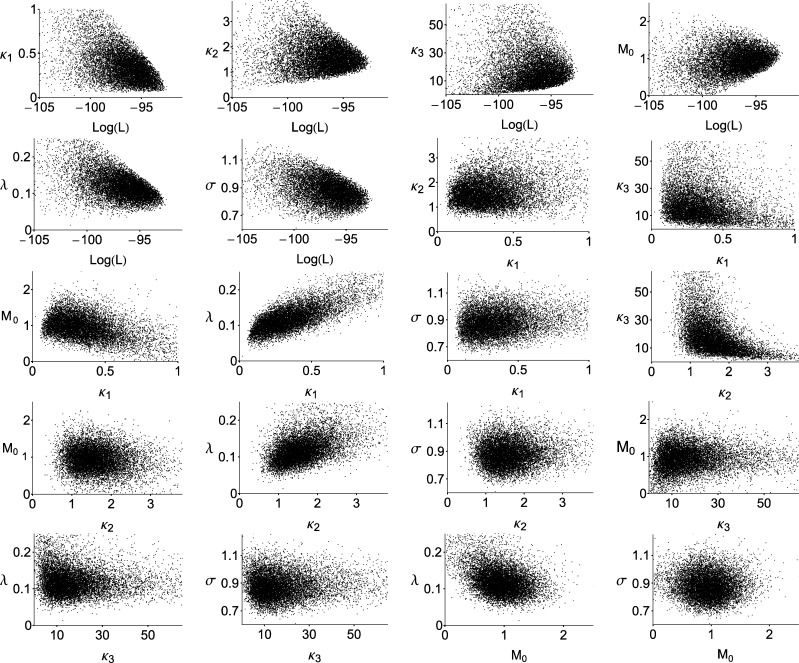
Posterior correlation between the parameters for the tetracyclin experiments

For the tetracyclin experiment, the maximum likelihood median and 95 % credibility intervals for the parameters are presented in Table 2. The best fit to the data is shown in Fig. 1. Histograms of the parameter distributions and the correlation between parameters are shown in the Appendix (Figs. 5 and 6). For all parameters but *κ*_3_, relatively tight posterior distributions are obtained. This implies that we have only little information about *κ*_3_, i.e. about the precise shape of the function that describes how the efflux pump production changes with the concentration of antibiotics in the cell. The MCMC updates can also be used to calculate how long it would take, after the *E. coli*’s are exposed to a certain antibiotic concentration for a long time, before the MIC-value of the strains are below a critical level. If this critical level is very high, even with antibiotic exposure, the MIC-value will remain below this level, and the time to reach an MIC-value below this critical level is zero. If the critical level is very low, even without antibiotic exposure, the MIC-value of the *E. coli* will exceed this critical level and we say that it takes infinitely long before the critical MIC-value is reached. If we take 8 mg/L as the cut-off value for tetracyclin resistance in *E. coli* (MIC_safe_), the median and 95 % confidence interval for the time *T*_*Q*_ are depicted as function of the external antibiotic concentration (*A*_0_) in Fig. 4. From this figure, we observe that after exposure to high doses of tetracyclin, it may take several days before the cells become susceptible to tetracyclin again, although the 95 % confidence upper bound becomes as high as 20 days.

**Table 2:** Maximum likelihood values, median and 95 % credibility interval (2.5 % quantile and 97.5 % quantile) for the tetracyclin and amoxicillin lab data

	Tetracyclin				Amoxicillin			
	MLE	Median	2.5 %	97.5 %	MLE	Median	2.5 %	97.5 %
log*L*	−92	−97	−101	−94	−81	−86	−91	−83
*λ*	0.07	0.12	0.07	0.19	0.13	0.49	0.13	0.92
*M*_0_	1.17	0.94	0.42	1.47	3.3	2.8	0.7	4.3
*σ*	0.80	0.87	0.75	1.04	0.8	0.9	0.8	1.1
*κ*_1_	0.09	0.33	0.12	0.77	4.4	4.9	3.9	6.1
*κ*_2_	1.34	1.55	0.90	2.79	3.5	5.6	0.4	66.9
*κ*_3_	19	15	4	51	0.04	2.1	0.13	519

### Amoxicillin experiments

**Fig. 7:**
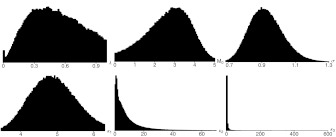
Histograms of the posterior distributions of the parameters for the tetracyclin experiments with a uniform prior on (0,1) for *λ*

**Fig. 8:**
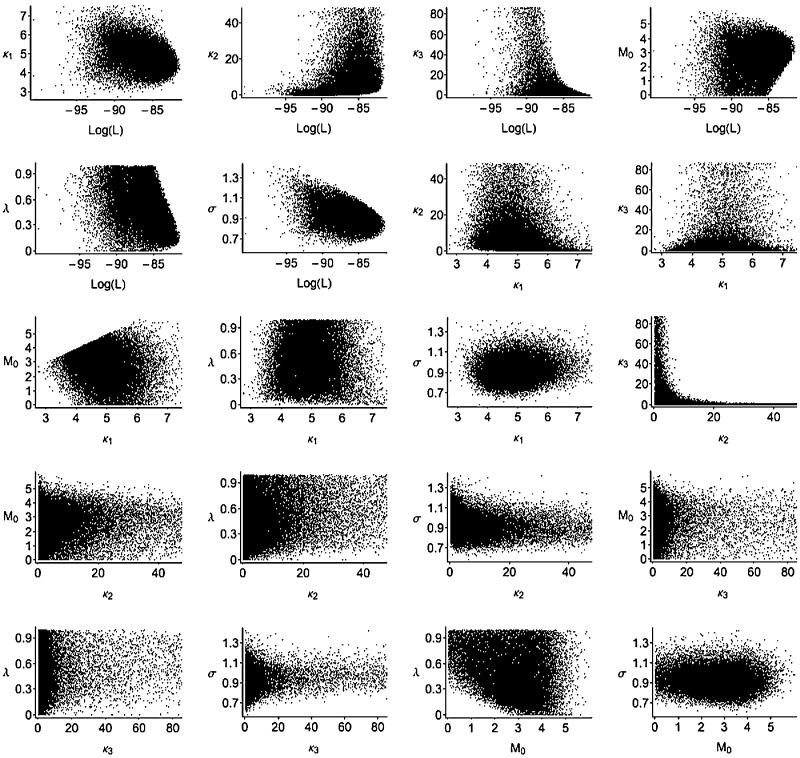
Posterior correlation between the parameters for the amoxicillin experiments with a uniform prior on (0,1) for *λ*

For the amoxicillin experiments, the best fit to the data (where the data from Fig. 2(g) were not used) is shown in Fig. 2. However, no proper a posteriori distributions for the parameters could be obtained. The reason is that the equilibrium MIC-value in absence of amoxicillin (*κ*_1_/*λ*) can be estimated, but there are not enough experiments to determine the parameters individually. More specifically, the data allow for very high values of *λ*, as long as the ratio (*κ*_1_/*λ*) stays more or less constant. This implies that the data do not exclude that the processes within a cell go extremely fast. Therefore, *T*_*Q*_ will be very short. To obtain an upper bound for *T*_*Q*_, we put a uniform prior (0,1) on the parameter *λ*. This way, we could obtain median and 95 % credibility intervals for the parameters (see Table 2). Histograms of the parameter distributions and the correlation between parameters are shown in the Appendix (Figs. 7 and 8). The time till the bacteria become susceptible again, after exposure to amoxicillin for a long period, is depicted in Fig. 4(b). However, we should stress that the experiment with an increasing amoxicillin concentration was not taken into account in this analysis. The time *T*_*Q*_ is not very sensitive to the choice for the value of MIC_safe_ (data not shown).

## Discussion

We have presented a mathematical model that exposes relationships between data from various experiments and, more importantly, allows us to extrapolate to situations for which no experimental results are available. This description is partly mechanistic and partly phenomenological. The estimation of the parameters is based on statistical methodology. The model predicts that relatively short antibiotic-free time periods are sufficient to re-obtain low MIC-values. For tetracyclin, the method works fine. For amoxicillin, we observed one experiment with inherited resistance, which we could not incorporate in our statistical analysis. As a result, there are too few experiments to perform a proper estimation of the rate at which non-inherited resistance to amoxicillin disappears. We would like to stress also that in order to obtain a model for which calculations were feasible, several simplifying assumptions had to be made. For instance, we do not explicitly consider the population size and the life cycle of individual cells and assume that we can use an average cell size during the life cycle of a bacterium and still obtain accurate results. Also, we assume that biological fluctuations are short-lived, in the sense that given the concentration of antibiotics to which the population of *E. coli* cells is exposed, two subsequent measurements of the MIC-value of the population are independent of each other.

When animals are slaughtered for meat production, these animals must not have been recently exposed to antibiotics in order to prevent that substantial amounts of antibiotics will be present in the human food chain. For instance, in the Netherlands, the minimal waiting time after exposure to antibiotics is 7 days for milk and eggs and 28 days for poultry meat unless explicitly stated otherwise (Boereboom et al. 2010). In this sense, the observation that non-inherited resistance disappears most likely within a period of several days is reassuring. However, this observation cannot be used to justify widespread use of antibiotics in the veterinarian sector as the conclusions from the experimental data are not directly applicable to experiments where production animals receive antimicrobial treatment. For instance, animals will carry many bacteria and also many *E. coli* strains, but not necessarily the strain used in the experiments. The mechanisms involved in competition between different bacterial species and between different *E. coli* strains are not well known. In particular, it is generally unknown how a competitive balance is influenced by the use of antibiotics. Moreover, the intestines of animals are not a well-mixed tank, different niches will be exposed to different concentrations of antibiotics, oxygen, nutrients, and so on. Finally, within a group of production animals several processes will occur simultaneously: Resistance-development, either due to mutation (as was observed in the enrofloxacin experiments) or due to a physiological/phenotypic change; selection between strains, e.g. for the strain with the highest initial MIC-value; transmission of bacteria between animals; transmission of mobile elements, e.g. plasmids, between bacteria. Hence, one should be careful to apply our conclusions directly to settings like the poultry industry. Still, we claim, the experiments, the model and our methodology can contribute to the discussion of what exactly characterizes a prudent use of antibiotics in modern agriculture.

## References

[CR1] Abel zur Wiesch P. A., Kouyos R., Engelstädter J., Regoes R. R., Bonhoeffer S. (2011). Population biological principles of drug-resistance evolution in infectious diseases. Lancet Infect. Dis..

[CR2] Boereboom, M., Fabri, T., Van Geijlswijk, I., Van Geloof, H., De Groot, S., Hagenaar, M., Verhoeven, G., & Mevius, D. (2010). Formularium pluimvee (in Dutch). Available at http://wvab.knmvd.nl/wvab/formularia/formularia.

[CR3] Bolla J.-M., Alibert-Franco S., Handzlik J., Chevalier J., Mahamoud A., Boyer G., Kieć-Kononowicz K., Pagès J.-M. (2011). Strategies for bypassing the membrane barrier in multidrug resistant Gram-negative bacteria. FEBS Lett..

[CR4] Davies J., Davies D. (2010). Origins and evolution of antibiotic resistance. Microbiol. Mol. Biol. Rev..

[CR5] Day T., Bonduriansky R. (2011). A unified approach to the evolutionary consequences of genetic and non genetic inheritance. Am. Nat..

[CR6] Drlica K., Hiasa H., Kerns R., Malik M., Mustaev A., Zhao X. (2009). Quinolones: action and resistance updated. Curr. Top. Med. Chem..

[CR7] Gilks W., Richardson S., Spiegelhalter D. (1996). Markov chain Monte Carlo in practice.

[CR8] Harbottle H., Thakur S., Zhao S., White D. (2006). Genetics of antimicrobial resistance. Anim. Biotechnol..

[CR9] Maragakis L., Perencevich E., Cosgrove S. (2008). Clinical and economic burden of antimicrobial resistance. Expert Rev. Anti. Infect. Ther..

[CR10] Mevius D., Wit B., Van Pelt W., Bondt N. (2010). MARAN monitoring of antimicrobial resistance and antibiotic usage in animals in The Netherlands in 2008.

[CR11] Schuurmans J., Nuri Hayali A., Koenders B., Ter Kuile B. (2009). Variations in mic value caused by differences in experimental protocol. J. Microbiol. Methods.

[CR12] Slama T. (2008). Gram-negative antibiotic resistance: there is a price to pay. Crit. Care.

[CR13] Van der Horst M., Schuurmans J., Smid M., Koenders B., Ter Kuile B. (2011). De novo acquisition of resistance to three antibiotics by *escherichia coli*. Microb. Drug Resist..

